# Aldo-keto reductase family 1 member C3 (*AKR1C3*) gene polymorphism (rs12529) is associated with breast cancer in Bangladeshi population: A case-control study and computational investigation

**DOI:** 10.1371/journal.pone.0318079

**Published:** 2025-06-09

**Authors:** Md. Akeruzzaman Shaon, Farzana Ansari, Zimam Mahmud, Sonia Tamanna, Md. Nazid Bin Ibrahim, Nazia Fairooz Alam, Md. Naiem Hossain, Md. Zakir Hossain Howlader

**Affiliations:** 1 Laboratory of Nutrition and Health Research, Department of Biochemistry and Molecular Biology, University of Dhaka, Dhaka, Bangladesh; 2 Laboratory of Nutritional, Gene and Human Disease, Department of Biochemistry and Molecular Biology, University of DhakaDhaka, Bangladesh; Instituto Nacional de Ciencias Médicas y Nutrición Salvador Zubiran: Instituto Nacional de Ciencias Medicas y Nutricion Salvador Zubiran, MEXICO

## Abstract

Breast cancer is defined as the unchecked growth of breast cells, with imbalances in prostaglandin and steroid hormone metabolism contributing to disease risk by altering prostaglandin types and forms (strong and weak) of steroid hormones. The AKR1C3 enzyme plays a key role in managing these metabolic processes. This study investigated the association between the *AKR1C3* gene polymorphism (rs12529) and the risk of developing breast cancer in Bangladeshi individuals. A case-control investigation was conducted with a total of 620 samples, involving 310 individuals diagnosed with breast cancer and 310 healthy subjects. Herein, DNA extraction was performed via an organic process, whereas genotyping was employed via the PCR‒RFLP technique. Statistical assessments were conducted to analyze the association of polymorphisms, while molecular dynamics simulation and diverse computational techniques were employed to anticipate the functional and structural impacts of the SNP. Our study discovered that the rs12529 polymorphism of the *AKR1C3* gene has an enhanced risk of susceptibility to breast malignancy (p = 0.016, OR = 1.97, 95% CI = 1.22 to 3.16 for the GG genotype in additive model 2). The recessive model (GG vs CC+CG) also showed an enhanced risk of susceptibility to breast malignancy (p = 0.0004, OR = 1.95, 95% CI =  1.40 to 2.73). In both premenopausal women and postmenopausal women, the GG genotype (for the recessive model) significantly increased breast cancer risk by 1.92-fold and 1.95-fold, respectively. However, no significant associations were observed regarding tumor grade or size in breast cancer development. In-silico analyses indicated that the H5Q (rs12529) mutation may decrease protein stability but is typically tolerated or functionally neutral. Molecular dynamics simulations revealed that H5Q leads to increased structural fluctuations and surface exposure, potentially causing the mutant AKR1C3 enzyme to operate differently from the wild type. In conclusion, rs12529 significantly increases the incidence of breast cancer in the population of Bangladesh. Computational analyses further revealed that the H5Q (rs12529) mutation in AKR1C3 leads to decreased stability and altered functional changes with notable conformational changes.

## 1. Introduction

Cancer is characterized by uncontrolled and continuous cell proliferation that spreads to different body parts, infecting healthy tissues and organs [1]. Among women, breast carcinoma is the most frequently diagnosed form of cancer. It is considered a critical global health concern and stands as the leading cause of cancer-related mortality among women [2]. The majority of breast tumors arise from the cells lining the ducts and are referred to as ductal carcinomas. In addition, lobular malignancies develop in the cells lining the lobules, while a small percentage grow in other tissues [3]. Global Cancer Observatory (GLOBOCAN) 2022 reported that breast cancer was the most prevalent form of cancer for women (https://gco.iarc.who.int/media/globocan/factsheets/populations/900-world-fact-sheet.pdf) representing 23.8% of newly identified cases of female cancer worldwide. Moreover, according to GLOBOCAN 2022 data, Bangladesh reported 12,989 new breast cancer cases and 6,162 deaths, placing the disease fourth in terms of incidence and sixth in mortality within the country (https://gco.iarc.who.int/media/globocan/factsheets/populations/50-bangladesh-fact-sheet.pdf).

Understanding the risk factors for breast cancer is essential for early screening, which significantly enhances the likelihood of successful treatment outcomes. Breast cancer is more prevalent in developed nations due to factors such as early onset of menstruation, delayed age at first childbirth, absence of children, obesity, alcohol consumption, insufficient physical activity, and limited breastfeeding [4]. The variation in the proteins involved in their activation or inactivation plays a key role in assessing an individual’s risk of developing cancer [5]. Furthermore, it is crucial to guide therapeutic strategies by locating genetic markers associated with breast cancer development, as personalized medication is possible with the help of genetic risk profiles [6].

In the exploration of the genetic landscape of breast cancer [7,8], it has become evident that a hereditary component plays a pivotal role in the disease etiology, accounting for 5-10% of all cases [9]. Central to this genetic framework are the BRCA1 and BRCA2 genes, in which pathogenic variants significantly increase breast cancer risk [10], and other genes, such as PALB2, TP53, PTEN, CDH1, CHEK2, and ATM, each contributing to the multifaceted nature of breast cancer susceptibility [11,12]. These genetic factors highlight the autosomal dominant inheritance patterns in most hereditary breast cancer cases and the intricate interplay between high-penetrance genes and numerous moderate-to-low penetrance genes across various populations. Furthermore, alterations in genes such as MYC, ERBB2, FGFR1, GATA3 [13], and AKR1C3 [14,15] in tumor cells signal their critical roles in the progression of early breast malignancies. Our target gene, the Aldo-keto reductase family 1 member C3 (AKR1C3) gene, has been reported to be highly expressed in breast malignancies and is associated with tumor invasiveness, aggression, and resistance to chemotherapy [16,17].

The Aldo-keto reductase 1C (AKR1C) superfamily gene comprises four closely related isoforms (AKR1C1-AKR1C4) [18,19]. The AKR1C3 enzyme is translated from the AKR1C3 gene located on chromosome 10 at position p15-p14. This gene spans approximately 13 kb and has nine exons. Transcripts typically range in size, with the most common being approximately 1.4 kb [20]. The AKR1C3 protein is approximately 37 kilodaltons in molecular mass and consists of 323 amino acids. The three-dimensional form of the protein has been elucidated, revealing a typical (α/β)_8_-barrel crease, a characteristic of the Aldo-keto reductase superfamily. The catalytic center contains a deeply buried NADP(H) cofactor binding region, which is essential for the enzyme’s reduction activity. The structures also provide insights into the substrate specificity and catalytic mechanism of these enzymes [21].

The AKR1C3 enzyme metabolizes a wide range of substrates [22]. It mainly acts as a 17-ketoreductase that changes the attraction of hormone molecules to their specific receptors. For instance, AKR1C3 transforms 5α-androstane-3,17-dione and Δ^4^-androstene-3,17-dione into 5α-dihydrotestosterone and testosterone, which increases their attraction to androgen receptors [23]. AKR1C3 converts progesterone and estrone into 20α-hydroxyprogesterone and 17β-estradiol, respectively, controlling their ability to bind to estrogen receptors and progesterone receptors [24,25]. AKR1C3 controls cell growth and specialization by acting as a prostaglandin F2 (PGF2) synthase [26]. The conversion of hormones by AKR1C3 can enhance the development of breast cancer cells. Initially, two AKR1C3 metabolites, 17β-estradiol and 20α-OHP, support the growth of breast tumor cells [27]. AKR1C3 also promoted the production of PGF2 products, which in turn supported the proliferation of breast tumor cells [26]. Furthermore, AKR1C3 is involved in reducing endogenous reactive aldehydic or ketonic chemicals and aiding in their removal via the phase I biotransformation activity of other enzymes [28]. AKR1C3 can also process various external substances, such as numerous chemotherapeutic agents, utilizing carbonyl reductase action, leading to detoxification and resistance to chemotherapy [19,29].

Many single nucleotide polymorphisms (SNPs) have been found in the coding and noncoding regions of the *AKR1C3* gene. Molecular epidemiology studies have established correlations among many of these SNPs and different illnesses. Nevertheless, these connections are present, and not all SNPs have functional consequences [30]. One of these SNPs is rs12529 which appears disproportionately in different ethnic backgrounds [31]. This evidence prompted the idea that the rs12529 variant may influence the advancement, intensity, or reaction to therapy differently in different populations in the case of illnesses where AKR1C3 has a crucial role [32–36]. Shiota *et al*. [33] reported that the rs12529 variant did not hinder the enzyme’s operation but may impact its physiological function by causing changes in gene expression, posttranslational modifications, or intracellular enzyme degradation. Again, a meta-analysis suggested that the altered allele (G) of rs12529 might be attributed to increased cancer risk in Asians [37]. In studies of the relationship between the rs12529 polymorphism and cancer, Lan Q *et al*. [35] reported that Chinese people who carry the rs12529 homozygous GG genotype had significantly increased susceptibility to lung cancer. Again, the occurrence of the rs12529 mutation was linked to alterations in physical well-being of life observed with androgen deprivation therapy (ADT) in prostate cancer, where the minor allele elicited a greater response. However, the impact of rs12529 (H5Q) on the process of androgen production still needs to be identified [36]. The rs12529 variant is a significant predictive factor when AKR1C3 inhibitors are created for clinical use [35]. The functional analysis showed that rs12529 (H5Q) had a *k*_*cat*_*/K*_*m*_ reduction ranging from 40 to 200 times for the 17-ketosteroid reduction of exemestane. Reduced production of the active metabolite 17β-dihydroexemstane (DHE) leads to increased resistance to exemestane drugs in the case of breast cancer [33].

The primary goal of this investigation was to compare a small group of healthy females to breast cancer patients in order to evaluate the importance of the C15G (rs12529) variant as a potential risk factor. Additionally, the study explored the structural and functional changes in the AKR1C3 enzyme caused by this polymorphism (rs12529) using computational techniques such as molecular dynamics simulation and molecular docking.

## 2. Methods and materials

### 2.1 Study participants

This was a population-based case-control study in which breast cancer patients were considered cases. Participants with a previous history of any cancer were excluded from the study, as were individuals currently undergoing chemotherapy or any other cancer treatment. Additionally, participants with severe comorbidities such as cardiovascular diseases, autoimmune disorders, or conditions that could significantly alter genetic expression or immune responses were excluded. The control group consisted of age-matched healthy individuals without any history of chronic diseases, ensuring that the study focused on genetic associations without the confounding effects of pre-existing conditions or treatments. This study included a total of 620 participants, comprising 310 individuals diagnosed with breast cancer (cases) and 310 age-matched healthy individuals as controls. The research received approval from the Ethical Review Committee of the Department of Biochemistry and Molecular Biology at the University of Dhaka (Ref. No. BMBDU-ERC/EC/23/014). Furthermore, we affirm that all procedures conducted during this study adhered to the applicable guidelines and regulations.

Participants were selected from the National Institute of Cancer Research & Hospital (NICRH) in Dhaka, Bangladesh, during the period from July 1, 2023, to December 30, 2023. They were diagnosed with breast carcinoma using mammography, breast ultrasound, biopsy, or magnetic resonance imaging (MRI). All participants were female. During the same timeframe, control subjects were recruited from the National Institute of Ear, Nose, and Throat (NIENT) in Dhaka, Bangladesh. These individuals, all female, had no history or signs of cancer. Both the case and the control groups were recruited from Dhaka and matched for age and sex to minimize bias. As Dhaka is the capital and the primary hub for medical treatment in Bangladesh, attracting patients from all regions of the country [37], and predictably, we have found people from all divisions of Bangladesh. Additionally, the socio-economic status of the controls closely mirrored that of the breast cancer patients, with the majority of both groups coming from lower-income backgrounds.

Informed written consent was obtained from all participants prior to sample collection. Participants were fully briefed on the study’s objectives, procedures, and potential risks and were informed of their right to withdraw without consequence. All data were anonymized to ensure confidentiality by removing personal identifiers and assigning unique study codes. Electronic data were stored in a password-protected file, while paper records were kept in a locked file cabinet. Data analysis was performed in aggregate to safeguard participants’ privacy further. Data on sociodemographic factors, such as age, height, weight, family income, living area, educational background, medical history, menstrual and reproductive history, and family history of cancer, were collected using a structured questionnaire. Participants were also asked to provide comprehensive details about their breast cancer history, including pathological tumor grade, tumor size, age at diagnosis, prescribed medications, total white blood cell count (WBC), erythrocyte sedimentation rate (ESR), and biomarker status for progesterone receptor (PR), estrogen receptor (ER), and human epidermal growth factor receptor 2 (HER2).

### 2.2 Sample collection

Trained phlebotomists drew 5.0 mL of venous blood from each participant using disposable syringes under strict aseptic conditions. The collected blood was transferred into vacutainer tubes containing ethylene diamine tetra acetic acid (EDTA). Plasma was separated by centrifuging the samples at 3,000 rpm for 15 minutes. Both the plasma and cellular components were stored at -20°C for future analysis.

### 2.3 Genotyping of rs12529 (C15G)

DNA was isolated from the fractions of cells using the organic extraction method. Genotyping of rs12529 was conducted through polymerase chain reaction-restriction fragment length polymorphism (PCR-RFLP). The PCR reactions were set up in a total volume of 15 µ L within a PCR tube containing 7.5 µ L of GoTaq G2 Green Master Mix (Promega Corporation), 5.05 µ L of nuclease-free water, 0.45 µ L of DMSO, 0.5 µ L each of forward and reverse primers, and 1 µ L of extracted DNA. The forward primer used was F (5′-TGCAATTTTCTCCACAGACCA-3′), and the reverse primer was R (5′-AAGCAGTACGTGACCATAGGA-3′). Details of the primer sequences are presented in S1 Table, while the PCR conditions are outlined in S2 Table.

**Fig 1:**
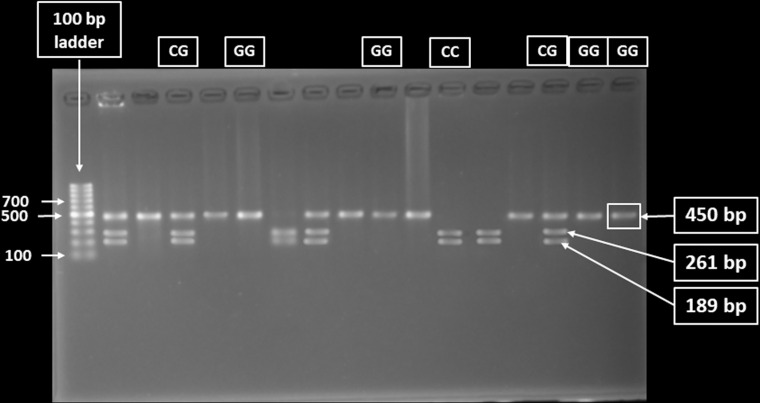
Representative restriction enzyme-digested products on a 2% agarose gel. The presence of 261 bp and 189 bp on (from left) the 7^th^, 12^th^, and 13^th^ wells indicates the existence of the homozygous wild-type C/C genotype. The 450 bp, 261 bp, and 189 bp in lanes 2^nd^, 4^th^, 8^th^, and 15^th^ indicate the existence of a heterozygous mutant C/G genotype. In comparison, the 450 bp on lanes 3^rd^, 5^th^, 6^th^, 9^th^, 10^th^, 11^th^, 14^th^, 16^th^ and 17^th^ indicate the homozygous mutant G/G genotype. The first lane (from left) contains a 100 bp DNA ladder.

The 450 bp PCR product was subjected to digestion using the AleI v2 restriction enzyme. The reaction, performed in a 15 μL volume at 37°C for 16 hours, produced two fragments of 189 bp and 261 bp in the presence of the reference C allele. For the mutant homozygous G/G genotype, no cleavage occurred, resulting in a single 450 bp fragment. The heterozygous C/G genotype yielded three fragments, measuring 450 bp, 261 bp, and 189 bp, respectively. The wild-type homozygous C/C genotype generated two fragments of 261 bp and 189 bp, as illustrated in Fig 1. To ensure reliable genotyping results, multiple quality control measures were implemented. Negative (no-template) controls were included in each PCR batch to detect contamination. Minimal PCR failures were reprocessed as needed. Duplicate PCRs were conducted for a subset of samples, and inconsistent replicates were re-analyzed to confirm results. PCR products were separated on 2% agarose gels and visualized under UV light. Two researchers independently performed genotyping results, with ambiguous results reviewed and retested for accuracy. Only samples with clear, reproducible results were included in the final analysis.

### 2.4 False-positive report probability (FPRP) analyses

The FPRP was determined to evaluate the reliability of the results [38]. A threshold of 0.2 was established for the FPRP, and prior probabilities of 0.25, 0.01, 0.001, and 0.0001 were used to identify an odds ratio (OR) of 1.5 associated with cancer risk. Results were deemed significant if their FPRP values were less than 0.2. The choice of an OR of 1.5 was based on standard practice in genetic epidemiology, where an OR of 1.5 is often used as a threshold for detecting moderate effects that are biologically plausible [39]. While this decision was not directly influenced by prior literature in this population, it aligns with typical thresholds for evaluating the significance of genetic associations [40].

### 2.5 Statistical analyses

We calculated the sample size using the G * Power program version 3.1.9.7 [41], [Type I error, α= 5% and predicted power, (1-β) =  80%]. The calculated sample size stated a number of at least 308 for each case and control group. Data analysis was conducted using GraphPad Prism (version 10.1.2) and the R programming language (version 4.1.2). Quantitative variables, such as age and BMI, were represented as the mean ±  standard deviation (SD), while categorical variables were described as percentages (%). Differences in the means of continuous variables were evaluated using unpaired t-tests, and relationships between categorical variables were analyzed with two-tailed chi-square tests. The link of *AKR1C3* gene rs12529 polymorphism with breast cancer was estimated by logistic regression according to four genotypic models (additive model 1, additive model 2, dominant model, and recessive model) and allele model using OR with 95% confidence intervals (CI). The Akaike information criterion (AIC) and Bayesian information criterion (BIC) are used to compare model fit while accounting for complexity. AIC rewards fit but penalizes excessive parameters to avoid overfitting [42], while BIC applies a more substantial penalty for complexity, particularly with larger sample sizes [43]. Based on these AIC and BIC values, the best-fit model was selected. The OR at the 95% CI was adjusted with age at menarche. A p-value of less than 0.05 indicated statistical significance in all analyses. The Bonferroni correction was applied in this study to control the risk of Type I errors due to multiple comparisons, the newly calculated p-value =  (observed p-value ×  number of observations). Adjusting the observed p-value enhances the reliability of results in analyses with numerous tests, such as genotypic models and subgroup analyses. This p-value correction method maintains the integrity of the findings by controlling the family-wise error rate (FWER) and ensuring that associations are not due to random [44]. The FPRP analyses were performed using the gap package of R programming language [45]. Hardy-Weinburg equilibrium (HWE) was made using the “SHEsisPlus” (http://shesisplus.bio-x.cn/SHEsis.html) web tool [46,47].

### 2.6 In silico analysis

Various in-silico analysis tools were utilized following the methodologies outlined in our previously published research [48–50]. For example, SIFT (Sorting intolerant from tolerant) (https://sift.bii.a-star.edu.sg) [51] and Polyphen-2 (Polymorphism phenotyping v2) (http://genetics.bwh.harvard.edu/pph2) [52] were utilized for the prediction of the effect of the SNPs on the function of the protein. PredictSNP (https://loschmidt.chemi.muni.cz/predictsnp/) [53], SNAP (Semi-HMM-based nucleic acid parser) [54], PhD-SNP (Predictor of human deleterious single nucleotide polymorphisms) (https://snps.biofold.org/phd-snp/phd-snp.html) [55], and MAPP (Multivariate analysis of protein polymorphisms) (http://mendel.stanford.edu/SidowLab/downloads/MAPP/index.html) [56] were used to estimate disease associations with the SNPs. MUpro (https://mupro.proteomics.ics.uci.edu/) [57] and INPS-MD (Impact of nonsynonymous mutations on protein stability - multidimension) (https://inpsmd.biocomp.unibo.it/inpsSuite/) [58] were used to predict protein stability due to polymorphisms. The HOPE (https://www3.cmbi.umcn.nl/hope/) database was utilized to analyze changes in proteins due to amino acid substitution [59]. SWISS-MODEL (https://swissmodel.expasy.org/) [60] was used to construct a 3D model of the mutant protein using the Q95JH6.1 template from the AlphaFold database (https://alphafold.ebi.ac.uk/) [61,62]. Swiss model assessment, PROSA (https://prosa.services.came.sbg.ac.at/prosa.php) [63], and ERRAT (https://saves.mbi.ucla.edu/) [64] were used to validate the 3D model. Using AutoDock Vina v1.2.0 [65], the binding energy of protein-ligand docking was calculated.

### 2.7 Molecular dynamics (MD) simulations

The GROningen MAchine for Chemical Simulations (GROMACS) (version 2020.6) was utilized for a 100 nanosecond molecular dynamics simulation [66] for the ligand-protein complexes. The simulated scenario employs the CHARMM36m force field. A water box was created around the protein surface with corners located 1 nm away utilizing the TIP3 water model. The necessary ions were utilized to balance the systems. A simulation lasting 100 ns was conducted under periodic boundary conditions and a temporal integration step of 2 fs after energy minimization, isothermal isochoric (NVT) equilibration, and system isobaric (NPT) equilibration. The trajectory data were analyzed using a snapshot interval of 100 picoseconds. The GROMACS program’s embedded rms, rmsf, gyrate, sasa, and h-bond packages were utilized to analyze the simulation’s root mean square deviation (RMSD), root mean square fluctuation (RMSF), radius of gyration (Rg), and solvent accessible surface area (SASA). Plots for the investigations as mentioned earlier were produced utilizing the ggplot2 program in RStudio. At the Bioinformatics Division of the National Institute of Biotechnology, high-performance simulation stations utilizing the Ubuntu 20.04.4 LTS operating system were used for all MD simulations.

## 3. Results

### 3.1 Patients’ demographic characteristics

**Fig 2:**
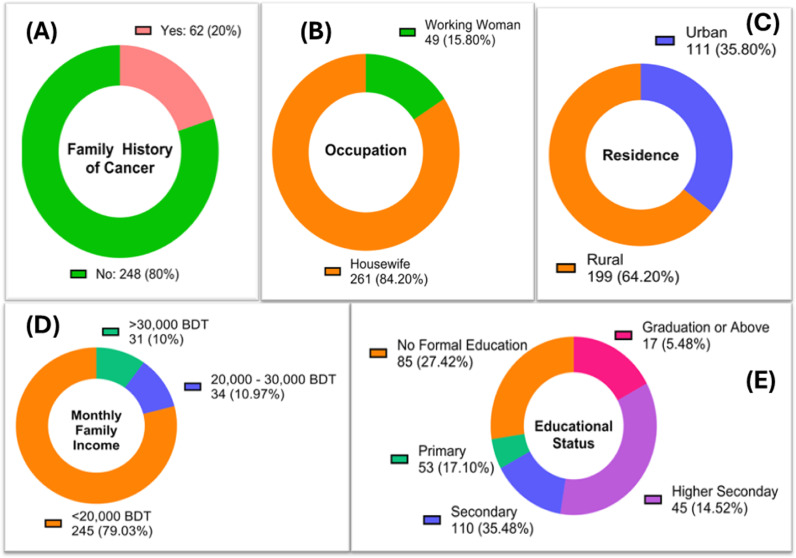
Demographic characteristics of the breast cancer patients enrolled in the study. (A) shows the patients’ family history of cancer, (B) shows the occupation of the patients, (C) represents the area of residence, (D) presents the range of family income of the patients, and (E) demonstrates the educational status of the patients.

Among the 310 patients included in this study, 80% had no previous family history of cancer. Most of the patients (84.20%) were housewives by profession. The majority of the patients (64.20%) resided in rural areas of Bangladesh, which is reflected by their family income. 79.03% of patients had a family income lower than 20,000 BDT per month, and 27.42% had no formal education, with 17.10% and 35.48% with primary and secondary education, respectively. The demographic characteristics are shown in Fig 2.

### 3.2 Baseline characteristics of the participants

Table 1 presents the study subjects’ baseline characteristics according to age, BMI, menstrual history, age at first menstruation, and pregnancy count. The results revealed significant differences between BC patients and healthy controls regarding age at menarche but not age, BMI, menstrual status, or number of pregnancies.

**Table 1:** Baseline characteristics of study subjects.

Variables		Case (n = 310) Mean ± SD	Control (n = 310) Mean ± SD	p-Value
Age (Year)		43.04 ± 10.49	43.27 ± 9.71	0.79 (ns)
BMI (kg/m^2^)		24.40 ± 4.60	23.94 ± 4.08	0.21 (ns)
		**n (%)**	**n (%)**	
Age group	< 30	19 (6.13)	24 (7.74)	0.076 (ns)
	30-50	240 (77.42)	250 (80.65)	
	> 50	51 (16.45)	36 (11.61)	
BMI group	≤ 25	183 (59.03)	194 (62.58)	0.37 (ns)
	> 25	127 (40.97)	116 (37.41)	
Menstrual status	Premenopausal	139 (44.84)	131 (42.26)	0.52 (ns)
	Postmenopausal	171 (55.16)	179 (57.74)	
Age at menarche	10-13	171 (55.16)	249 (80.32)	**<0.0001 (s)**
	14-17	139 (44.84)	61 (19.68)	
Number of Pregnancies	< 3	181 (58.38)	173 (55.81)	0.52 (ns)
	≥ 3	129 (41.62)	137 (44.19)	

ns = not significant; s = significant; Bold values indicate statistically significant.

### 3.3 Clinicopathological data of breast cancer patients

As shown in Table 2, the clinicopathological data showed that 52.58% of patients were diagnosed with cancer after 40 years of age. In this study, nearly all patients (98.06%) were diagnosed with invasive ductal carcinoma (IDC), while only 6 patients had invasive lobular carcinoma (ILC). For hormone receptor status, all patients (100%) were ER + and PR + , with 67.42% being HER2 + . Furthermore, 67.42% of patients had a tumor with a diameter greater than 2 cm (T2 and T3), and 64.84% of patients had grade 2 (G2) tumors.

**Table 2:** The clinicopathological information of the patients involved in this study.

Variables (n = 310)		Number (n)	Percentage (%)
Age at diagnosis	≤ 40	147	47.42
	>40	163	52.58
Histological type	IDC	304	98.06
	ILC	6	1.94
Receptor status	ER +	310	100
	ER-	0	0
	PR +	310	100
	PR-	0	0
	HER2 +	209	67.42
	HER2-	101	32.58
Tumor size	T1 (≤2 cm)	101	32.58
	T2 (>2 and ≤ 5 cm)	197	63.55
	T3 (>5 cm)	12	3.87
Grading of tumor	G1	49	15.81
	G2	201	64.84
	G3	60	19.35

### 3.4 Genotypic distribution of *AKR1C3* rs12529 polymorphism and the risk of developing breast cancer

The genotype frequencies among control subjects were 19.40%, 44.80%, and 35.80% for homozygous wild type (Hz wild, CC), heterozygous variant (Ht variant, CG), and homozygous variant (Hz variant, GG), respectively. Among the patients, the frequencies were 14.50%, 33.50%, and 51.90% for Hz wild type (CC), Ht variant (CG), and Hz variant (GG), respectively.

The association analysis according to the additive model 1, additive model 2, dominant model, and recessive model is shown in Table 3. Additive model 2 was found to increase the statistically significant risk of breast cancer development (p = 0.016) in the case of the homozygous variant GG. The homozygous variant GG significantly increased the risk of breast cancer by 1.97 times (p = 0.016, OR = 1.97, 95% CI = 1.22 to 3.16). The recessive model showed a 1.95-fold increase in the risk of developing breast cancer, and the risk was found to be statistically significant (p = 0.0004, OR = 1.95, 95% CI = 1.40 to 2.73). The recessive model was identified as the best-fit model based on its lowest AIC (314) and BIC (322.86) values, indicating it provided the most parsimonious explanation of the data. These values were lower than those of the other models, which provided a statistically significant association, suggesting the recessive model best represents the relationship between AKR1C3 rs12529 genotype and breast cancer risk in our cohort. These findings support including the recessive model as the most appropriate for further analysis.

**Table 3:** Frequency distribution of rs12529 (C15G) genotypes in breast cancer patients and control subjects.

Genetic Model	Genotype/ Allele	Controls (n = 310) n (%)	Cases (n = 310) n (%)	OR (95% CI)	p-Value^a^	p-Value^b^	AIC	BIC
Additive Model 1 (CG vs CC)	CC	60 (19.40)	45 (14.50)	1.00 (Ref.)	>0.99 (ns)	>0.99 (ns)	304	312.86
	CG	139 (44.80)	104 (33.50)	1.01 (0.62 to 1.63)				
Additive Model 2 (GG vs CC)	CC	60 (19.40)	45 (14.50)	1.00 (Ref.)	0.004 (s)	0.016 (s)	324	332.86
	GG	111 (35.80)	161 (51.90)	1.97 (1.22 to 3.16)				
Dominant Model (CG+GG vs CC)	CC	60 (19.40)	45 (14.50)	1.00 (Ref.)	0.11 (ns)	0.44 (ns)	284	292.86
	CG+GG	250 (80.70)	265 (85.50)	1.43 (0.92 to 2.23)				
Recessive Model (GG vs CC+CG)	CC+CG	199 (64.20)	149 (48.10)	1.00 (Ref.)	0.0001 (s)	0.0004 (s)	**314**	**322.86**
	GG	111 (35.80)	161 (51.90)	1.95 (1.40 to 2.73)				
Allele Frequency (G vs C)	C	259 (41.77)	194 (31.29)	1.00 (Ref.)	0.0001 (s)	---	---	---
	G	361 (58.23)	426 (68.71)	1.58 (1.25 to 1.99)				

OR: odds ratio, 95% CI: 95% confidence interval, Ref.: Reference, ns: not significant, s: significant. AIC and BIC values in bold indicate the best model. ORs were adjusted with age at menarche.

^p^-Value^a^: p-value of chi-square test.

p-Value^b^: Bonferroni corrected p-value.

The allele frequencies were 0.4177 and 0.5823 for wild-type (C) and altered (G) alleles, respectively, among the control subjects. On the other hand, the frequencies were 0.3129 and 0.6871 for the wild-type and altered alleles, respectively, among the patients. The allele frequency analysis revealed that the risk of developing breast cancer was 1.58 times greater in G allele carriers (p = 0.0001, OR = 1.58, 95% CI = 1.25 to 1.99) than in those with the reference C allele.

**Fig 3:**
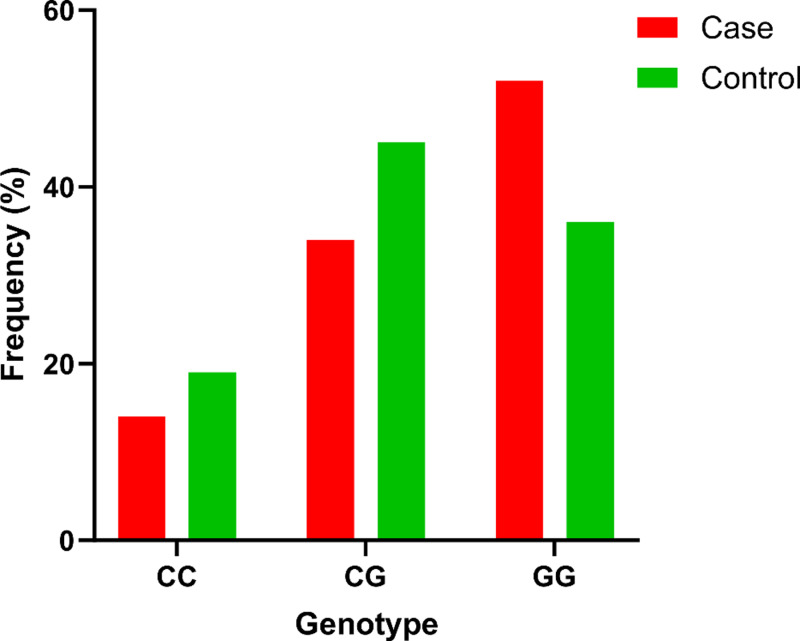
Genotypic distribution of rs12529 in the study subjects. The GG genotype was dominant in the case (51.90%) group. The least frequently observed genotype was CC for both the case (14.50%) and control (19.40%) groups.

The genotype distribution of rs12529 in the study participants is shown in Fig 3.

### 3.5 False-positive report probability (FPRP) results

Table 4 presents the FPRP values along with the statistical power for findings related to the AKR1C3 rs12529 polymorphism. Based on the FPRP analysis, the additive model 2, recessive model, and allelic model demonstrated a statistically significant association of rs12529 with breast cancer, with FPRP values of 0.10, 0.005, and 0.0001, respectively, at a prior probability threshold of 0.25.

**Table 4:** FPRP values for the association between rs12529 and breast cancer risk.

Genetic Model	OR (95% CI)	p-Value^b^	Statistical power^a^	Prior probability				
				0.25	0.10	0.01	0.001	0.0001
Additive Model 1 (CG vs CC)	1.01 (0.62 to 1.63)	>0.99 (ns)	0.95	0.75	0.90	0.99	1.00	1.00
Additive Model 2 (GG vs CC)	1.97 (1.22 to 3.16)	**0.016 (s)**	0.13	**0.10**	0.26	0.79	0.97	1.00
Dominant Model (CG+GG vs CC)	1.43 (0.92 to 2.23)	0.44 (ns)	0.58	0.37	0.64	0.95	0.99	1.00
Recessive Model (GG vs CG+CC)	1.95 (1.40 to 2.73)	**0.0004 (s)**	0.063	**0.005**	0.014	0.14	0.61	0.94
Allelic Model (G vs C)	1.58 (1.25 to 1.99)	**0.0001 (s)**	0.33	**0.0009**	0.003	0.03	0.24	0.76

Note: The results of the FPRP analysis are highlighted in bold when the FPRP value is below 0.2.

^a^Statistical power was determined based on the number of observations within the subgroup, along with the OR and p-values presented in this table.

^b^p-Value: Bonferroni corrected p-value.

### 3.6 Frequency distribution of rs12529 (C15G) genotypes in the study subjects according to menopausal status

Table 5 presents the distribution of the AKR1C3 rs12529 genotype in patients and control subjects according to menopausal status.

**Table 5:** The distribution of rs12529 (C15G) genotypes among participants categorized by menopausal status.

Menopausal status	Genetic model	Genotype/ Allele	Controls n (%)	Cases n (%)	OR (95% CI)	p-Value^b^
**Pre-menopausal** Controls 131 Case 139	Additive Model 1	CC	25 (19.08)	20 (14.39)	1 (Ref.)	>0.99 (ns)
		CG	59 (45.04)	47 (33.81)	0.99 (0.49 to 2.07)	
	Additive Model 2	CC	25 (19.08)	20 (14.39)	1 (Ref.)	0.24 (ns)
		GG	47 (35.88)	72 (51.80)	1.92 (0.97 to 3.92)	
	Dominant Model	CC	25 (19.08)	20 (14.39)	1 (Ref.)	>0.99 (ns)
		CG+GG	106 (80.92)	119 (85.61)	1.40 (0.74 to 2.60)	
	Recessive Model	CC+CG	84 (64.12)	67(48.20)	1 (Ref.)	**0.036 (s)**
		GG	47 (35.88)	72 (51.80)	1.92 (1.18 to 3.09)	
**Post-menopausal** Controls 179 Cases 171	Additive Model 1	CC	35 (19.55)	25 (14.62)	1 (Ref.)	>0.99 (ns)
		CG	80 (44.69)	57 (33.33)	0.99 (0.53 to 1.84)	
	Additive Model 2	CC	35 (19.55)	25 (14.62)	1 (Ref.)	0.12 (ns)
		GG	64 (35.76)	89 (52.05)	1.95 (1.06 to 3.52)	
	Dominant Model	CC	35 (19.55)	25 (14.62)	1 (Ref.)	0.88 (ns)
		CG+GG	144 (80.45)	146 (85.38)	1.42 (0.81 to 2.49)	
	Recessive Model	CC+CG	115 (64.24)	82 (47.95)	1 (Ref.)	**0.008 (s)**
		GG	64 (35.76)	89 (52.05)	1.95 (1.28 to 3.02)	

Ref.: Reference, ns: not significant, s: significant. p-values in bold indicate statistically significant.

p-Value^b^: Bonferroni corrected p-value.

In the case of premenopausal women, the GG genotype carriers showed a 1.92 times increasing risk of the development of breast malignancy (p = 0.036, OR = 1.92, 95% CI =  1.18 to 3.09). The other models (additive model 1, additive model 2, and dominant model) didn’t show any statistically significant association with the risk of breast cancer development. Again, while analyzing the data of postmenopausal women, the same GG genotype carriers showed a 1.95 times enhanced risk of breast malignancy development (p = 0.008, OR = 1.95, 95% CI =  1.28 to 3.02). The rest of the three models (additive model 1, additive model 2, and dominant model) didn’t present any statistically significant association with the risk of breast cancer development.

### 3.7 Association of rs12529 (C15G) with tumor grade and size in breast cancer patients

In the patient group, polymorphism associations with tumor size and grade were analyzed. The results are presented in Tables 6 and 7. The alternative allele (G) showed no significant association with tumor size or grade.

**Table 6:** Distribution of rs12529 (C15G) genotypes in patients with different tumor grades.

Genotype	Tumor grade		p-Value	OR (95%CI)
	G1 (n = 49) n (%)	G2 + G3 (n = 261) n (%)		
CC	11 (22.45)	34 (13.03)	0.09 (ns)	1 (Ref.)
CG+GG	38 (77.55)	227 (86.97)		1.93 (0.86 to 4.11)

ns: not significant, OR: odds ratio, Ref.: reference, 95% CI: 95% confidence interval.

**Table 7:** Distribution of rs12529 (C15G) genotypes in patients with different tumor sizes.

Genotype	Tumor size		p-Value	OR (95%CI)
	T1 (≤2 cm) (n = 101) n (%)	T2 + T3 (>2 cm) (n = 209) n (%)		
CC	19 (18.81)	26 (12.44)	0.14 (ns)	1 (Ref.)
CG+GG	82 (81.19)	183 (87.56)		1.63 (0.87 to 3.10)

ns: not significant, OR: odds ratio, Ref.: reference, 95% CI: 95% confidence interval.

### 3.8 Assessment of constancy in genotype frequency

Table 8 shows the results of the Hardy‒Weinberg equilibrium (HWE) test, which was used to evaluate the consistency of genetic frequency in the study subjects. According to the p-values, the genotypes of rs12529 were in the HWE for the healthy controls (p = 0.397), but in the case of breast cancer patients, the genotypes of the rs12529 deviated from the HWE.

**Table 8:** Hardy‒Weinberg equilibrium test of rs12529 in the study subjects.

Study Subjects	χ^2^ Statistic	p-Value
Case	16.08	0.0003
Control	1.84	0.397

### 3.9 In silico analysis

#### 3.9.1 Analysis of functional consequences of rs12529 (H5Q).

Various in-silico tools were utilized to assess the impact of mutations on protein function. All tools classified the H5Q mutation as either tolerated or neutral. However, MUpro and INPS-MD predicted a reduction in the stability of the associated protein due to this mutation. The detailed predictions are summarized in Table 9, while the scores from different web-based tools are provided in S3 Table.

**Table 9:** Functional effects of rs12529 (H5Q) on the AKR1C3 protein.

Name of tool	Prediction
SIFT	Tolerated
Polyphen2	Benign
PhD-SNP	Neutral
PredictSNP	Neutral
MAPP	Neutral
SNAP	Neutral
MuPro	Decrease stability
INPS-MD	Decrease stability

Additionally, the HOPE server was used to analyze several characteristics impacted by amino acid substitution. The H5Q mutation introduced an amino acid with distinct differences in size, charge, and hydrophobic properties. This alteration in amino acid composition led to a decrease in interactions and the disruption of hydrogen bonding. A summary of the key findings from the HOPE analysis can be found in S4 Table.

#### 3.9.2 Homology modeling.

The complete three-dimensional structure of the human AKR1C3 protein was obtained from the AlphaFold database. The FASTA sequence (UniProt ID: P42330) was then utilized to model the 3D structure of the mutant AKR1C3 protein (H5Q) using Q95JH6.1.A (AlphaFold ID) as the template. The predicted structure was assessed using the SWISS-MODEL structure evaluation tool, ProSA-Web, and ERRAT, all of which indicated satisfactory results and high-quality models. The evaluation scores from these tools are detailed in S5 Table.

#### 3.9.3 Protein-ligand docking.

The affinity between the native form of the protein-ligand complex and its mutant variant varies. Specifically, the interaction between the wild-type AKR1C3 protein and its ligand NADPH had a binding energy of -10.8 kcal/mol. This energy shifts to -9.2 kcal/mol when examining the interaction of the mutant H5Q AKR1C3 with NADPH.

#### 3.9.4 Molecular dynamics (MD) simulation.

##### 3.9.4.1 Root mean square deviation (RMSD) of the wild type and mutant (H5Q) AKR1C3 protein.

**Fig 4:**
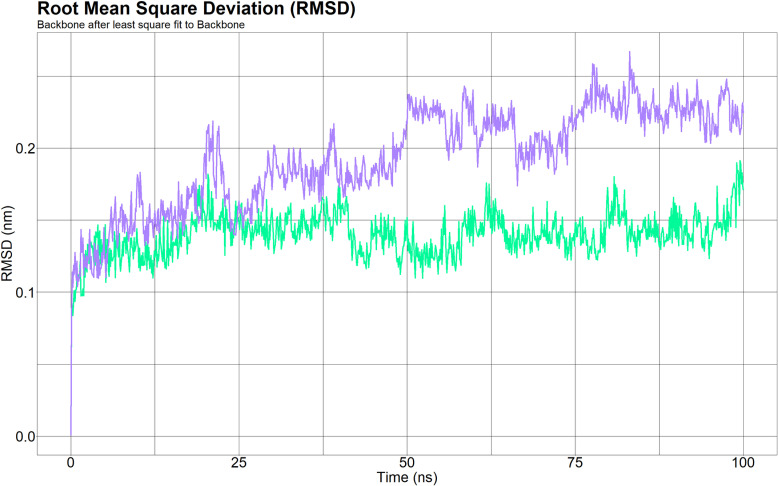
Root mean square deviation of wild-type AKR1C3 (green line) and mutant (H5Q) AKR1C3 (purple line) proteins. The pattern of conformational change in wild-type AKR1C3 (green line) was significantly different from that in mutant AKR1C3 (purple line).

The stability of the protein-ligand complex was assessed through RMSD analysis. Variations in RMSD values indicate the extent of conformational adjustments in the protein triggered by the interaction with a ligand. In this context, the RMSD value for the native form of AKR1C3, in complex with NADPH as the ligand, was approximately 0.15 nm. Conversely, the RMSD for the mutant variant of AKR1C3, which also complexed with NADPH, was approximately 0.2 nm. This finding demonstrated a distinct pattern of conformational changes between the wild-type and mutant proteins. The wild-type AKR1C3-NADPH complex (green line) and mutant AKR1C3-NADPH complex (purple line) are depicted in Fig 4.

##### 3.9.4.2 Root mean square fluctuation (RMSF) of the wild-type and mutant (H5Q) AKR1C3 protein.

**Fig 5:**
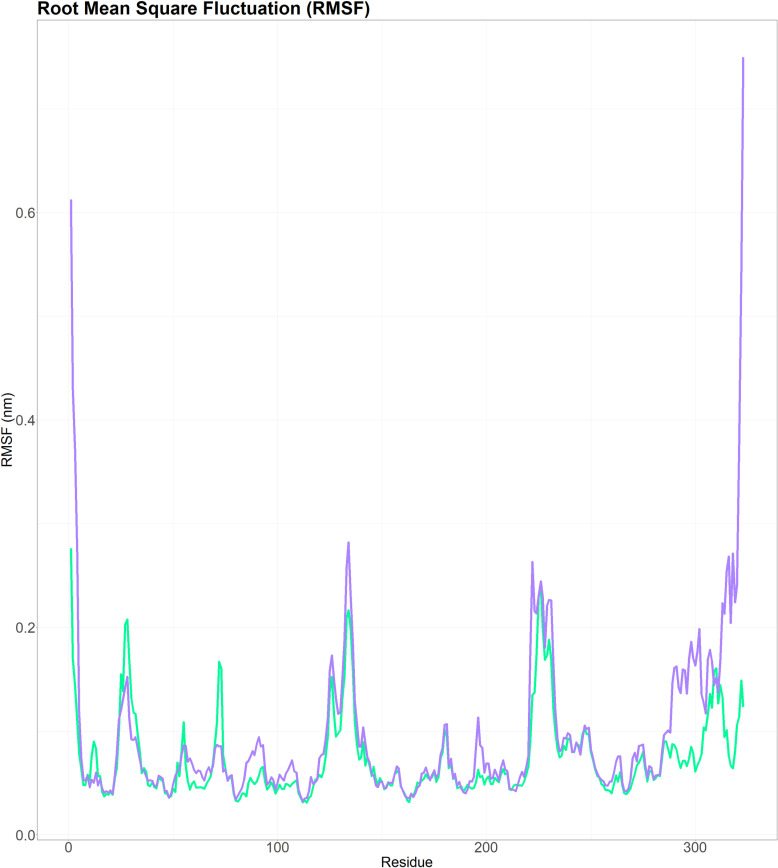
Root mean square fluctuations of the wild-type (green line) and mutant (H5Q) (purple line) proteins of AKR1C3 (complexed with the ligand NADPH). The mutant version exhibited distinct RMSF patterns, with two major peaks in the first 100 amino acids absent in the mutant and five significant peaks beyond the 100^th^ amino acid that were higher than those observed in the wild type.

The RMSF graph shows the mobility of wild-type (green line) AKR1C3 (complexed with the ligand, NADPH) and mutant (purple line) AKR1C3 (complexed with the ligand, NADPH). From the 1^st^ amino acid to the 100^th^ amino acid of wild-type AKR1C3 (complexed with the ligand, NADPH), two peaks corresponding to RMSF were observed, whereas no peak corresponding to mutant AKR1C3 (complexed with the ligand, NADPH) was detected. Five more peaks were detected from the 100th amino acid to the end of the mutant AKR1C3 protein than those in the wild-type AKR1C3 protein (Fig 5).

##### 3.9.4.3 The radius of gyration of wild and mutant (H5Q) AKR1C3 complexed with the ligand NADPH.

**Fig 6:**
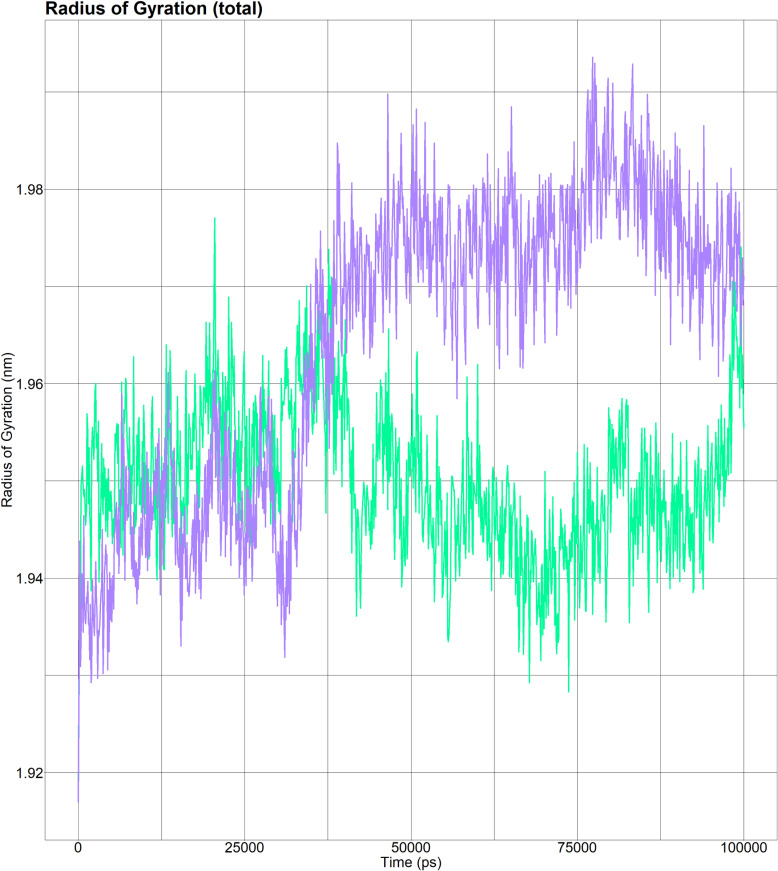
The radius of gyration of wild-type and mutant (H5Q) AKR1C3 (both complexed with the ligand, NADPH). The Rg value of wild-type (green line) AKR1C3 was relatively stable during the simulation.

The Rg of the mutant (purple line) AKR1C3 (complexed with the ligand NADPH) was relatively unstable. In contrast, the Rg of the wild type (green line) AKR1C3 (complexed with the ligand NADPH) was relatively stable during the simulation (Fig 6).

##### 3.9.4.4 Solvent accessible surface area (SASA) of wild-type and mutant (H5Q) AKR1C3 complexed with the ligand NADPH.

**Fig 7:**
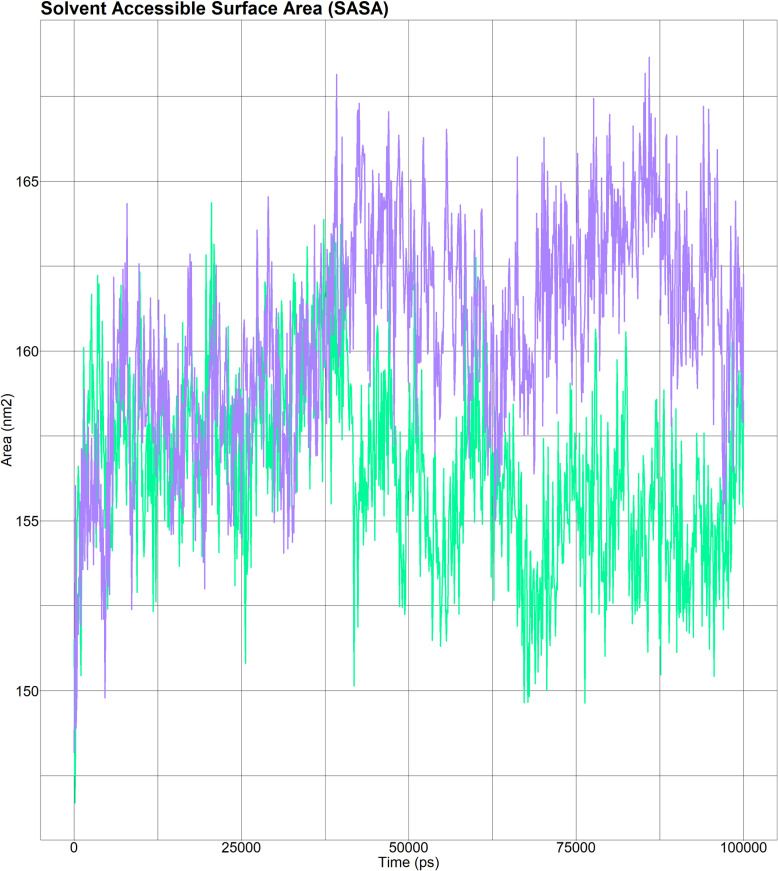
The solvent accessible surface area (SASA) of wild-type (green line) AKR1C3 (complexed with the ligand, NADPH) was lower than that of the mutant (purple line) AKR1C3 (complexed with the ligand, NADPH).

The SASA values of wild-type (green line) AKR1C3 (complexed with the ligand, NADPH) and mutant (purple line) AKR1C3 (complexed with the ligand, NADPH) significantly differed throughout the simulation (Fig 7). The wild-type variant had a lower SASA value than the mutant type.

## 4. Discussion

Breast cancer is the most prevalent form of cancer affecting women. It is relatively common in countries with substantial economies; although it is currently modest, the rate is increasing in emerging nations. This disease is the result of a mix of hereditary and environmental causes. Although the precise mechanism of breast cancer development is not yet known, several proven risk factors, including early menarche, late menopause, age at first childbirth, infertility, and genealogy, have been previously identified [67]. Furthermore, exposure to environmental variables such as ionizing radiation and carcinogens from chemicals has been linked to increased chances of developing breast cancer [68].

AKR1C3 displays pluripotency through varying levels of 3-keto-, 17-keto-, and 20-ketosteroid reductase activity [69]. Its 17-ketosteroid reductase function distinguishes the enzyme as 17β-HSD (17β-hydroxysteroid dehydrogenase) type 5, which is responsible for generating testosterone through an intracrine process and is different from the androgenic 17β-HSD (type 3 17β-hydroxysteroid dehydrogenase) [70] and the estrogenic 17β-HSD (type 1 17β-hydroxysteroid dehydrogenase) [71,72]. Therefore, any nonsynonymous mutation that increases the activity of the 17β-HSD type 5 form of AKR1C3 can increase testosterone availability. This excessive testosterone can further be converted into more potent estradiol by aromatase, which can initiate more growth responses in breast cells. In addition, this mutation can also alter the amount of other androgens and estrogens by affecting the structural and functional activities of androgenic 17β-HSD (type 3 17β-hydroxysteroid dehydrogenase) and estrogenic 17β-HSD (type 1 17β-hydroxysteroid dehydrogenase).

The 3-ketosteroid reductase function of AKR1C3 is represented by the enzyme 3α-HSD type 2, whose function is to convert the potent steroid hormone 5α-dihydrotestosterone (5α-DHT) into weak 5α-androstane-3α,17β-diol [18,69,73]. Thus, it reduces the amount of testosterone and its potent derivative 5α-dihydrotestosterone (5α-DHT). Therefore, any kind of nonsynonymous mutation that decreases the activity of the 3α-HSD type 2 form of AKR1C3 can inhibit the conversion of 5α-dihydrotestosterone (5α-DHT) into the weak 5α-androstane-3α,17β-diol. Therefore, more 5α-dihydrotestosterone (5α-DHT) and its immediate previous form, testosterone, can be further converted into the more potent estradiol by aromatase, which can initiate more growth responses in breast cells. AKR1C3 also acts as a prostaglandin (PG) F2α synthase and transforms PGH2 to prostaglandin F2α (PGF2α) and prostaglandin D2 (PGD2) to 11β-prostaglandin F2α (11β-PGF2α) [74,75]. PGF2α epimers function as ligands for the FP receptor, stimulating cell proliferation pathways [20]. Therefore, any gain-of-function mutation can promote the production of 11β-PGF2α and result in excessive breast growth and other cells.

This study aimed to evaluate the relationship between the rs12529 polymorphism in the AKR1C3 gene and the risk of breast cancer within the Bangladeshi population. Furthermore, the effects of this polymorphism on the AKR1C3 protein were analyzed using various in-silico tools and molecular dynamics simulations. The rs12529 (H5Q) variant shows varying frequencies across different ethnic groups [31]. This polymorphism has been well studied for its relation with various types of malignancies, e.g., prostate cancer [32], lung cancer [31], and bladder cancer [76], in different populations. However, rs12529 is comparatively less studied for its association with breast cancer. Its association with breast cancer risk has been studied in only 2 populations, i.e., the French and Canadian populations, and there was no significant association between rs12529 and breast cancer risk [77]. In South Asia, the association between rs12529 and breast cancer has not yet been studied. In Bangladesh, various studies have explored the associations between breast cancer and different gene polymorphisms, including rs13181 of ERCC2, rs2276466 of ERCC4, rs80357713 and rs80357906 of BRCA1, rs11571653 of BRCA2, rs1136201 of HER2, rs1042522 of TP53, rs16260 of CDH1, rs25487 of XRCC1, rs861539 of XRCC3, as well as rs1219648, rs2420946, and rs2981582 of the FGFR2 gene [78–82]. Consequently, this study represents the first to investigate the link between rs12529 of the AKR1C3 gene and breast cancer across the entire South Asian population.

This study examined the relationship between the AKR1C3 gene rs12529 (H5Q) polymorphism and breast cancer in a specific group of people from the Bangladeshi community. We analyzed the AKR1C3 genotypes of 620 individuals, including 310 healthy individuals and 310 breast cancer patients. Both groups were similar in age, BMI, menstrual status, and number of pregnancies but were significantly different in age at menarche (Table 1). This study revealed a positive correlation between this genetic variation and the development of breast cancer in Bangladeshi individuals.

In our study, the AKR1C3 rs12529 genotypes and genotype frequencies varied significantly between the two groups, and the frequency of the G allele was greater in BC patients than in healthy individuals (Table 3), although our results are not consistent with the results of Plourde *et al*. (2009) [77]. We observed a significantly increased risk of breast cancer due to rs12529 in additive model 2 (GG vs CC) and recessive model (GG vs CC+CG). The risk of developing breast cancer was 1.97-fold (OR = 1.97; 95% CI = 1.22 to 3.16; p = 0.004) greater in breast cancer patients with the GG genotype (in the case of additive model 2). Women with the GG genotype (recessive model) were 1.95 times more likely to develop breast cancer, and this difference was statistically significant (OR = 1.95; 95% CI = 1.40 to 2.73; p = 0.0004). When stratified against menopausal status, we found a statistically significant association between rs12529 and the risk of breast cancer development in both pre-menopausal women and post-menopausal women. The GG genotype (recessive model) significantly increased breast cancer risk by 1.92- and 1.95-fold, respectively, in the case of premenopausal and postmenopausal women.

This association between the AKR1C3 polymorphism (rs12529) and breast cancer risk in the Bangladeshi population is consistent with findings from other Asian populations, where the G allele of rs12529 has been found to be linked to an increased risk of cancer [35]. However, studies in European populations, such as those in France and Canada, have not found significant associations between rs12529 and breast cancer risk [77]. These discrepancies may be due to differences in allele frequencies, genetic backgrounds, or environmental factors across ethnic groups. In South Asia, the G allele appears to be more prevalent, suggesting that this polymorphism may have a more substantial effect in these populations. Comparing these results with global studies, especially considering the varying allele frequencies, will enhance our understanding of the genetic risk factors for breast cancer and may inform personalized approaches to prevention and treatment.

In this study, we found no significant association between the rs12529 (H5Q) polymorphism and tumor grade or size in breast cancer patients. This suggests that while rs12529 may influence breast cancer susceptibility, it may not directly affect tumor aggressiveness or other clinicopathological features. These findings highlight the complex nature of breast cancer biology, where genetic, environmental, and hormonal factors shape tumor progression. Previous studies [35,77] have not explored the potential link between rs12529 (H5Q) and tumor grade or size in breast cancer.

In the case of HWE test results, the p-value of 0.397 indicates that the genotype frequencies are consistent with HWE for the healthy control subjects. This suggests that, in the control group, the alleles at the rs12529 locus segregate randomly and that no factors such as selection, mutation, or non-random mating influence these frequencies significantly. In contrast, the breast cancer patients exhibit a significant deviation from HWE, as evidenced by a p-value of 0.0003 and a high chi-square statistic (χ² =  16.08). This deviation could indicate that one or more evolutionary forces are acting on the rs12529 locus within this group. One potential explanation for the deviation from HWE in cases could be population stratification or substructure within our study population. While we attempted to recruit participants from a homogenous population, subtle genetic substructures might still exist. Another factor that could contribute to the deviation from HWE is genotyping errors. Moreover, the possibility of sampling bias due to the relatively small sample size may contribute to the deviation from HWE. Overall, deviations from HWE may occur due to natural selection related to breast cancer, population stratification, or genotyping errors. However, the latter is less likely, given that the control group is in equilibrium.

The utilization of various in-silico tools to assess the effect of the H5Q mutation on protein function yielded insights into its tolerance or neutral nature, with MUpro and INPS-MD specifically revealing a decrease in protein stability. The HOPE server analysis further delineated the impact of the mutation, emphasizing alterations in amino acid properties that led to disrupted interactions and hydrogen bonds. Notably, the mutation affected the binding energy of the ligand NADPH, indicating an alteration in affinity for the mutant AKR1C3. These findings collectively suggest that while the H5Q mutation is biochemically tolerated, it induces subtle yet significant changes in the structural and functional landscape of the protein, potentially influencing its biological role and therapeutic targeting strategies. These findings are consistent with the results reported by Shiota *et al*. [34], who concluded that the rs12529 variation could impact the physiological roles of AKR1C3 by modifying its transcriptional process, posttranslational changes, or intracellular degradation of proteins.

### Molecular dynamics simulation

A molecular dynamics simulation study on rs12529 revealed notable differences in the behavior of the AKR1C3 enzyme between the wild type and its mutant form when both were bound to NADPH. The RMSD analysis showed that the wild-type AKR1C3-NADPH complex had lower RMSD values, suggesting minor conformational shifts relative to the mutant form. This observation indicated that ligand binding induces more pronounced structural changes in the mutant protein. Furthermore, RMSF data indicated altered flexibility patterns in the mutant AKR1C3, potentially affecting its function or structure. The introduction of the H5Q mutation was found to destabilize AKR1C3, as evidenced by fluctuating Rg values, suggesting conformational changes and decreased protein compactness. Additionally, the mutant exhibited a greater SASA, indicating greater surface exposure, which could have implications for enzymatic activity or interaction capabilities, highlighting the structural and functional impact of the rs12529 mutation on AKR1C3.

Molecular dynamics simulations and in-silico analyses provided key insights into the functional impact of the rs12529 (H5Q) mutation in AKR1C3. The mutation was found to reduce protein stability, increase structural fluctuations, and alter ligand binding affinity. These changes may impair the enzyme’s ability to metabolize key steroids, such as testosterone and dihydrotestosterone (DHT), which are involved in estrogen biosynthesis via the aromatase pathway. Given estrogen’s role in breast cancer proliferation, this alteration could enhance the production of potent estrogens, such as estradiol, promoting cancer progression. Additionally, the mutation may affect AKR1C3’s role in prostaglandin synthesis, further contributing to tumorigenesis. Previous functional analyses also showed that rs12529 (H5Q) decreases the k_*cat*_/K_*m*_ for 17-ketosteroid reduction by AKR1C3, supporting its potential impact on breast cancer [33]. Our findings highlight AKR1C3 as a potential therapeutic target in breast cancer, particularly for individuals with H5Q mutation. Targeting AKR1C3 with enzyme inhibitors or steroid metabolism modulators could offer personalized treatment strategies. Further research on AKR1C3 variants and their impact on estrogen synthesis and prostaglandin pathways is essential for understanding their role in breast cancer pathology.

Our investigation revealed limitations that must be considered when interpreting our results. First, the sample size was not large. Furthermore, we focused on only a single variant; considering the multitude of mutations that can impact the way a gene functions, it seems insufficient to make conclusive findings. Future studies require larger sample sizes, different populations, and additional genetic variations. There is a significant opportunity for further investigations into the expression rates of AKR1C3 variants in BC patients and for examining the specific underlying biological mechanisms. Consequently, future investigations should consider these findings to uncover this connection.

Overall, our study identified a genetic variant that is significantly associated with breast cancer risk. This finding supports the role of genetic predisposition in the pathogenesis of breast cancer, highlighting specific gene variants and pathways that may be involved in the initiation and advancement of the illness. Moreover, our findings have important implications for developing personalized medicine approaches to breast cancer. By identifying genetic markers associated with increased risk, we can improve risk stratification and early detection strategies. This can lead to more tailored screening programs and potentially earlier diagnosis, which is crucial for improving patient outcomes. Additionally, understanding the role of these genes in breast cancer pathogenesis opens up possibilities for the development of targeted therapies aimed at specific molecular pathways, which could enhance treatment efficacy and reduce side effects.

In conclusion, we found an association between an AKR1C3 gene polymorphism (rs12529) and breast malignancy in Bangladeshi individuals. The frequency of the G allele was notably greater in breast cancer individuals than in control subjects. Thus, the GG genotype is considered a risk factor, and the CC genotype is a molecular marker for reducing breast cancer incidence. In-silico analyses and MD simulations suggested that the H5Q mutation in AKR1C3 results in decreased stability and increased structural fluctuations, indicating a potentially tolerable or neutral effect on function despite significant conformational changes compared to those of the wild type. Therefore, genotyping of the AKR1C3 (rs12529) gene could be a biomarker for the early diagnosis of breast cancer and could aid in personalized medicine as a novel therapy for breast carcinoma.

## Supporting information

S1 Fig Uncropped raw gel image of Fig 1. (PDF)

S1 Table Primers used to amplify target regions. (PDF)

S2 Table PCR conditions used to amplify the target regions. (PDF)

S3 Table Scores from different tools for the analysis of functional consequences of the target SNPs. (PDF)

S4 Table A depiction of the highlights of the HOPE predictions. (PDF)

S5 Table Evaluation scores of the predicted protein models. (PDF)

## Abbreviations

AKR1C3: Aldo-ketoreductase family 1 member C3
BC: Breast cancer
SNP: Single nucleotide polymorphism
DHE: 17β-dihydroexemstane
ESR: Erythrocyte sedimentation rate
PR: Progesterone receptor
ER: Estrogen receptor
HER2: Human epidermal growth factor receptor type 2
EDTA: Ethylenediaminetetraacetic Acid
PCR-RFLP: Polymerase chain reaction-restriction fragment length polymorphism
rpm: revolution per minute
RMSD: Root mean square deviation
RMSF: Root mean square deviation
Rg: Radius of gyration
SASA: Solvent accessible surface area
BMI: Body mass index
AIC: Akaike information criterion
BIC: Bayesian information criterion
OR: Odds ratio
95% CI: 95% confidence interval
Ref.: Reference
ns: not significant
s: significant
