# Supplementary material for: Aldo-keto reductase family 1 member C3 (AKR1C3) gene polymorphism (rs12529) is associated with breast cancer in Bangladeshi population: A case-control study and computational investigation
Source: PLoS One. 2025 Jun 9;20(6):e0318079. doi: 10.1371/journal.pone.0318079 (PMC12148162; doi:10.1371/journal.pone.0318079)
Supplement: S1 Fig — (PDF) [file pone.0318079.s001.pdf]

S1_raw_image (Without labelling): Uncropped gel image. Representative restriction enzyme-digested products on a 2% agarose gel.

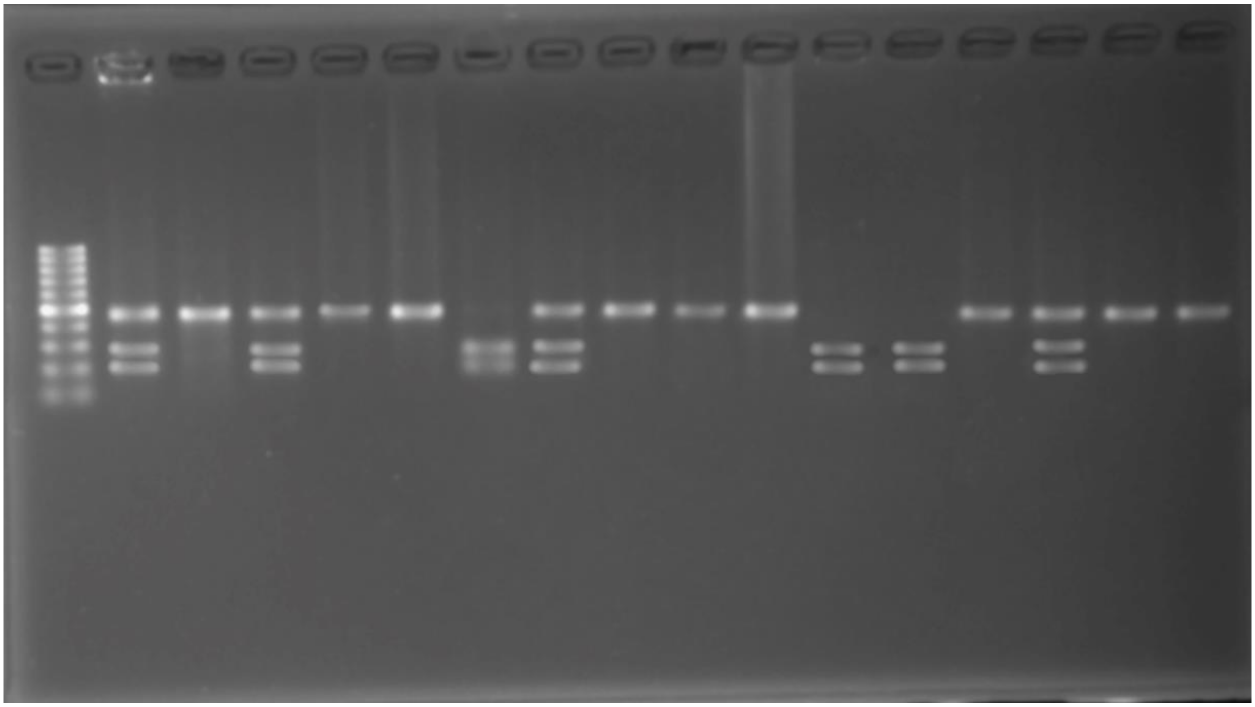

S1_raw_image (With labelling): Uncropped gel image. Representative restriction enzyme-digested products on a 2% agarose gel.

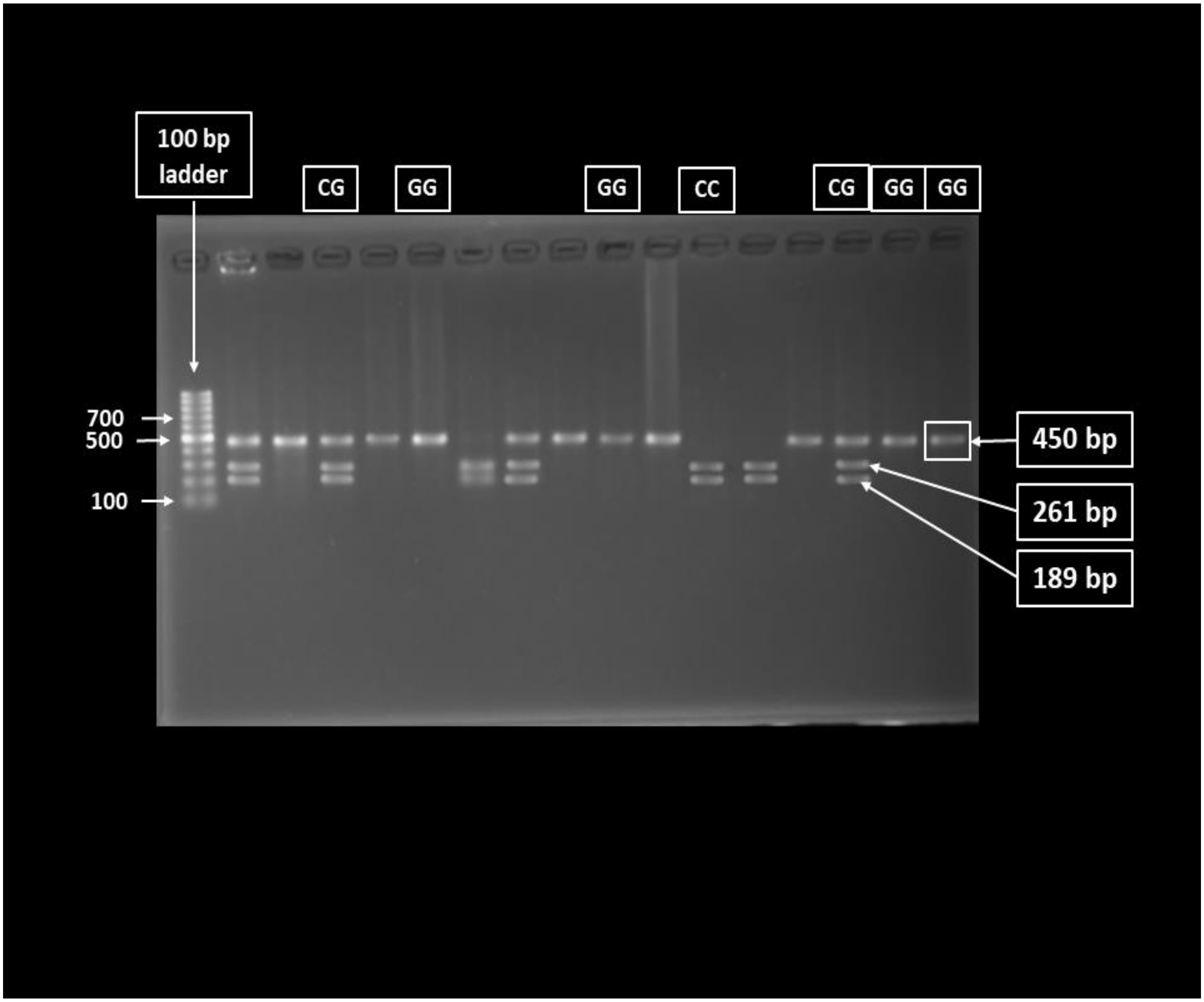

Note: Fig 1 was generated from the S1_raw_image.
