# Supplementary material for: Aldo-keto reductase family 1 member C3 (AKR1C3) gene polymorphism (rs12529) is associated with breast cancer in Bangladeshi population: A case-control study and computational investigation
Source: PLoS One. 2025 Jun 9;20(6):e0318079. doi: 10.1371/journal.pone.0318079 (PMC12148162; doi:10.1371/journal.pone.0318079)
Supplement: S1 Table — (PDF) [file pone.0318079.s002.pdf]

S1 Table. Primers used to amplify target regions.

Target	Direction	Sequence	Length (bp)	T_m (°C)	Product size (bp)
rs12529 (C15G)	Forward (5′-3′)	TGCAATTTTCTCCACAGACCA	21	59.2	450
	Reverse (5′-3′)	AAGCAGTACGTGACCATAGGA	21	59.4	

FP: forward primer; RP: reverse primer; bp: base pair.
