# Supplementary material for: Aldo-keto reductase family 1 member C3 (AKR1C3) gene polymorphism (rs12529) is associated with breast cancer in Bangladeshi population: A case-control study and computational investigation
Source: PLoS One. 2025 Jun 9;20(6):e0318079. doi: 10.1371/journal.pone.0318079 (PMC12148162; doi:10.1371/journal.pone.0318079)
Supplement: S2 Table — (PDF) [file pone.0318079.s003.pdf]

S2 Table. PCR conditions used to amplify the target regions.

T_m (°C)	PCR Conditions					
	95°C (Minutes)	Thermal cycle (40 cycles)			72°C (Minutes)	4°C (Seconds)
		95°C (seconds)	59°C (seconds)	72°C (seconds)		
59	5	45	42	95	5	Holding
