# Supplementary material for: Aldo-keto reductase family 1 member C3 (AKR1C3) gene polymorphism (rs12529) is associated with breast cancer in Bangladeshi population: A case-control study and computational investigation
Source: PLoS One. 2025 Jun 9;20(6):e0318079. doi: 10.1371/journal.pone.0318079 (PMC12148162; doi:10.1371/journal.pone.0318079)
Supplement: S3 Table — (PDF) [file pone.0318079.s004.pdf]

S3 Table. Scores from different tools for the analysis of functional consequences of the target SNPs.

SNP	Tool	Score
rs12529	SIFT	0.25
	Polyphen2	0.001
	PhD-SNP	0.89
	PredictSNP	0.83
	MAPP	0.79
	SNAP	0.71
	MuPro	-0.53
	INPS-MD	-0.277
