# Supplementary material for: Aldo-keto reductase family 1 member C3 (AKR1C3) gene polymorphism (rs12529) is associated with breast cancer in Bangladeshi population: A case-control study and computational investigation
Source: PLoS One. 2025 Jun 9;20(6):e0318079. doi: 10.1371/journal.pone.0318079 (PMC12148162; doi:10.1371/journal.pone.0318079)
Supplement: S4 Table — (PDF) [file pone.0318079.s005.pdf]

S4 Table. A depiction of the highlights of the HOPE predictions.

Difference in Size	Difference in charge	Conservation	Difference in hydrophobicity	Disrupt hydrogen bond
Yes	Yes	Not conserved	Yes	Yes
