# Supplementary material for: Aldo-keto reductase family 1 member C3 (AKR1C3) gene polymorphism (rs12529) is associated with breast cancer in Bangladeshi population: A case-control study and computational investigation
Source: PLoS One. 2025 Jun 9;20(6):e0318079. doi: 10.1371/journal.pone.0318079 (PMC12148162; doi:10.1371/journal.pone.0318079)
Supplement: S5 Table — (PDF) [file pone.0318079.s006.pdf]

S5 Table. Evaluation scores of the predicted protein models.

Models	Ramachandran favored region	ERRAT score	PROSA Z score
Wild type model	93.70%	97.43	-11.52
H5Q substitution model	93.40%	97.12	-11.20
